# High Tomato and Tomato Product Consumption is Protective Against the Decline in Handgrip Strength Among Japanese Adults: The Oroshisho Study

**DOI:** 10.2188/jea.JE20170029

**Published:** 2018-09-05

**Authors:** Zhongyu Ren, Cong Huang, Haruki Momma, Yufei Cui, Kaijun Niu, Shota Sugiyama, Masakazu Nanno, Ryoichi Nagatomi

**Affiliations:** 1 Department of Medicine and Science in Sports and Exercise, Tohoku University Graduate School of Medicine, Sendai, Japan; 2 Division of Biomedical Engineering for Health and Welfare, Tohoku University Graduate School of Biomedical Engineering, Sendai, Japan; 3 Department of Epidemiology, School of Public Health, Tianjin Medical University, Tianjin, China

**Keywords:** consumption of tomato and tomato product, handgrip strength, Japanese adults

## Abstract

**Background:**

There is scarce epidemiological evidence regarding the relationships of the consumption of different types of vegetables or fruits with change in skeletal muscle strength. We prospectively examined the relationships among Japanese adults, using handgrip strength to assess skeletal muscle strength.

**Methods:**

A 3-year study was carried out with 259 Japanese adults who were 22–68 years of age. The frequency of consumption of different types of vegetables or fruits were obtained using a validated self-administered dietary history questionnaire. Handgrip strength was measured with a handheld digital Smedley dynamometer.

**Results:**

After adjustment for confounding factors, the mean change in handgrip strength in participants stratified according to the level of tomato and tomato product consumption at baseline were −3.2 (95% confidence interval [CI], −4.0 to −2.3) for <1 time/week, −2.7 (95% CI, −3.6 to −1.8) for 1 time/week, −1.6 (95% CI, −2.5 to −0.8) for 2–3 times/week, and −1.7 (95% CI, −2.8 to −0.7) for ≥4 times/week, (*P* for trend = 0.022). However, the significant relationships of consumption of other types of vegetables and different types of fruits with change in handgrip strength were not observed.

**Conclusion:**

Higher consumption of tomato and tomato product at baseline was significantly associated with reduced decline in handgrip strength among Japanese adults over a 3-year follow-up period. This study suggests that consumption of tomato and tomato product could be protective against the decline in skeletal muscle strength associated with aging.

## INTRODUCTION

It is well known that aging leads to numerous anatomical and physiological degenerative changes, which can adversely affect physical function and skeletal muscle strength. Epidemiological studies have confirmed that lower skeletal muscle strength may predict all-cause mortality^1^ as well as several chronic diseases, including metabolic syndrome,^2^ type 2 diabetes mellitus,^3^ and cardiovascular disease.^4^ Thus, we considered that identification of easily modifiable determinants of decline in skeletal muscle strength is essential to the development of an effective preventive strategy.

Although the exact biological mechanisms involved in decline in skeletal muscle strength are not fully understood, enhanced oxidative stress may play an important role in individuals with lower skeletal muscle strength.^5^ In recent years, several studies have reported that dietary antioxidants, in particular, vitamin C, vitamin E, carotenoids, and lycopene, can help to protect against the decline in skeletal muscle strength with aging. Two cross-sectional studies showed that higher levels of dietary vitamin C^6^ and vitamin E^7^ were each independently associated with higher skeletal muscle strength in older women. In addition, a 6-year follow-up survey among older adults showed that those with lower plasma carotenoid levels are at a higher risk of declining skeletal muscle strength.^8^ However, lycopene has a stronger antioxidant ability to quench singlet oxygen and scavenge free radicals. Indeed, the antioxidant capacity of lycopene is 100 times that of vitamin E and 2 times that of other carotenes.^9^ Therefore, although lycopene may have a more favorable influence on the decline in skeletal muscle strength as compared to other antioxidants, the relationship between the consumption of dietary lycopene and decline in skeletal muscle strength has not been revealed.

Thus, we designed a 3-year longitudinal study to examine the relationship of the frequency of tomato and tomato product consumption, the richest dietary sources of lycopene, with change in handgrip strength among Japanese adults. To characterize the unique impact of consuming tomato and tomato product, fruit that are also rich in a large number of phytochemical antioxidants, we also examined the relationships of consumption of other types of vegetables and different types of fruits with change in handgrip strength.

## METHODS

### Study population

The Oroshisho study was carried out from 2008 to 2011 as continuous cross-sectional studies of 1,253 individuals working at the Sendai Oroshisho Center, which includes more than 120 small and medium size organizations in Sendai, Japan. A detailed study design has been previously published.^10^ Briefly, we recruited 1,253 individuals at baseline, who each received an annual health examination. Of these, 1,154 individuals agreed to participate and gave informed consent for analysis of their data. Of these, 617 individuals were excluded due to the following incomplete data at baseline (occupation, *n* = 6; drinking frequency, *n* = 73; metabolic syndrome, *n* = 5; adiponectin, *n* = 3; high-sensitivity C-reactive protein [hs-CRP], *n* = 1; physical activity [PA], *n* = 11; tomato and tomato product consumption, *n* = 1; handgrip strength, *n* = 517). We also excluded 278 individuals due to missing data on handgrip strength during the 3-year follow-up period. A total of 259 individuals (201 males and 58 females) were included in the study. Ethics approval was obtained from the Institutional Review Board of the Tohoku University Graduate School of Medicine.

### Dietary frequency assessment

All study participants completed a brief self-administered dietary history questionnaire (BDHQ) that assessed intake of 75 principal food items, with specified consumption frequency to assess the usual consumption of tomato and tomato product (tomato; tomato ketchup; stewed tomato; or tomato stew), other types of vegetables (green-leaf vegetables; cabbage and Chinese cabbage; carrot and pumpkin; Japanese white radish [daikon] and turnips; and onion, burdock, and lotus root), different types of fruits (citrus fruit; strawberries, persimmon and kiwifruit; apple and banana), and other dietary components during the preceding month. Individuals answered these questions using seven possible responses as follows: 0, <1, 1, 2–3, 4–6, 7, or ≥14 times/week. Based on the distribution of frequency, the frequency of consumption of tomato and tomato product and other types of vegetables was divided into four categories: <1 time/week, 1 time/week, 2–3 times/week, and ≥4 times/week; the frequency of consumption of different types of fruits was divided into three categories: 0 times/week, <1 time/week and ≥1 time/week. To calculate the frequency of combined or separated vegetable and fruit consumption, the monthly consumption frequency of each item was calculated and summed to create a new frequency of combined or separated vegetable and fruit consumption, and then these consumptions was converted into the weekly consumption frequency. Based on the distribution of frequency, combined or separated vegetable and fruit consumption were re-categorized as <4 times/week; 4–6 times/week, and ≥7 times/week. Daily nutrient intake, including total energy, total protein, calcium, and vitamin D were calculated using an ad hoc computer program for the BDHQ, with reference to the 5^th^ edition of the Standard Tables of Food Composition in Japan. The reproducibility and validity of the BDHQ have been described in detail elsewhere.^11^

### Handgrip strength measurement

Handgrip strength was measured using a handheld digital Smedley dynamometer (TKK 5401; Takei Scientific Instruments Co., Ltd., Niigata, Japan). All individuals were told to adjust dynamometer width for optimal hand comfort, and to relax their arm in a standing and stationary position. Each individual made two attempts using each hand with a brief interval between trials. The highest of all four handgrip strength measures was used as a representative value of skeletal muscle strength. The change in handgrip strength was calculated as: handgrip strength at follow-up − handgrip strength at baseline.

### Relevant covariant

Height and weight were measured using standard protocols. Body mass index (BMI) was calculated as weight (kg) divided by height squared (m^2^). Blood samples were drawn from the antecubital vein of seated subjects in the morning, following overnight fasting. Metabolic syndrome was defined according to the criteria of the American Heart Association Scientific Statement of 2009 for persons of Asian ethnicity (including Japanese).^12^ Serum adiponectin was measured using a specific sandwich enzyme-linked immunosorbent assay (Otsuka Pharmaceutical, Tokyo, Japan), and the intra- and inter-assay coefficients of variation were <10%. The concentration of serum hs-CRP was measured using N-latex CRP-2 (Siemens Healthcare Japan, Tokyo, Japan). Demographic variables and lifestyle factors were assessed using a self-administered questionnaire. Variables and factors assessed by the questionnaire included: sex (males or females), age (continuous variable), occupation (desk work or not), smoking status (never, former or current) and sleep duration (6–8 hr or not). Drinking frequency (7 times/week, 1–6 times/week or none) was also assessed using the BDHQ.^11^ Levels of PA were assessed using the International Physical Activity Questionnaire.^13^ The total weekly PA was calculated as follows: (daily hours of walking × days per week with walking × 3.3) + (daily hours of moderate intensity activity × days per week with moderate-intensity activity × 4.0) + (daily hours of vigorous activity × days per week with vigorous activity × 8.0). The total weekly PA was calculated as metabolic equivalents (METs·h/week).

### Statistical analysis

All statistical analyses were performed with SPSS (version 20.0; SPSS, Chicago, IL, USA). For the baseline characteristics of subjects, continuous variables are expressed as means and 95% confidence intervals, and categorical variables are expressed as percentages. Continuous data with skewed distributions, as determined using the Kolmogorov-Smirnov test, were logarithmically transformed. Analysis of covariance (ANCOVA) and logistic regression analysis was used to compare the baseline characteristics of the categorized group, after adjustment for sex and age. The categories of frequency for consumption of tomato and tomato product were considered the independent variables, and baseline variables were considered dependent variables.

ANCOVA was also used to estimate the change in handgrip strength based on the categories of consumption of tomato and tomato product, and other types of vegetables and different types of fruits with adjustment for age (continuous variable) at baseline (model 1). Model 2 was further adjusted for demographic, lifestyle, and clinic variables, such as: sex (categorical variable: males or females), occupation (categorical variable: desk work or not), smoking status (categorical variable: never, former, or current), drinking frequency (categorical variable: 7 times/week, 1–6 times/week or none), PA (continuous variable), sleep duration (categorical variable: 6–8 hr or not), BMI (continuous variable), metabolic syndrome (categorical variable: yes or no), hs-CRP (continuous variable), serum adiponectin (continuous variable), and handgrip strength (continuous variable) at baseline. Model 3 was further adjusted for daily nutrient intake and different types of vegetables and fruits consumption, such as: total energy (continuous variable), total protein (continuous variable), calcium (continuous variable), vitamin D (continuous variable), and mutual other types of vegetables (continuous variable) or mutual other types of fruits consumption (continuous variable) at baseline.

We also explored in more detail whether or not combined or separated vegetable and fruit consumption have a protective effect on decline in handgrip strength. Thus, we used multivariate adjusted models to assess the relationships between combined or separated vegetable and fruit consumption, and change in handgrip strength. Two-sided *P* values <0.05 were considered significant.

## RESULTS

The participants’ baseline characteristics, according to categories of consumption of tomato and tomato product are shown in Table 1. The higher frequency categories for consumption of tomato and tomato product contained a lower proportion of males (*P* for trend = 0.004), and higher average age than other frequency categories (*P* for trend = 0.005). Individuals with a higher frequency of consumption of tomato and tomato product also consumed more other types of vegetables (all *P* for trend <0.001), and citrus fruit (*P* for trend: 0.034), strawberries, persimmon and kiwifruit (*P* for trend: 0.008), apple and banana (*P* for trend: 0.026) and had higher consumption levels of total energy, total protein, calcium and vitamin D (all *P* for trend ≤0.005).

**Table 1.** Sex- and age-adjusted subject’s baseline characteristics according to frequency of consumption of tomatoes and tomato products^a^

Participants (*n* = 259)	Categories of tomato and tomato product consumption				*P* for trend^b^

	<1 time/week	1 time/week	2–3 times/week	≥4 times/week	
	(*n* = 70)	(*n* = 62)	(*n* = 70)	(*n* = 57)	
Mean intake, g/day	3.6	11.3	27.5	64.6	—
Grip strength (at baseline), kg	40.9 (39.5, 42.3)	42.5 (41.0, 44.0)	40.3 (39.0, 41.7)	40.2 (38.6, 41.8)	0.220
Grip strength (at follow-up), kg	37.6 (36.2, 39.0)	39.5 (38.1, 41.0)	38.9 (37.5, 40.3)	38.6 (37.0, 40.2)	0.505
**Demographic characteristics**					
Sex (males), %	82.9	85.5	77.1	63.2	0.004
Age, years	43.6 (41.3, 46.0)	44.3 (41.8, 46.8)	45.1 (42.7, 47.4)	48.8 (46.2, 51.5)	0.005
Occupation (desk work), %	44.3	37.1	45.7	63.2	0.369
**Lifestyle characteristics**					
Smoking status					
Current, %	50.0	54.8	42.9	33.3	0.135
Former, %	7.1	19.4	17.1	14.0	0.135
Drinking frequency					
7 times/week, %	28.6	32.3	34.3	22.8	0.734
1–6 times/week, %	71.4	67.7	65.7	77.2	0.734
PA, MET·h·week^−1^	51.0 (34.1, 68.0)	34.0 (16.0, 52.0)	33.4 (16.5, 50.3)	33.0 (13.8, 52.1)	0.190
Sleep duration (6–8 hr), %	57.1	38.7	57.1	63.2	0.210
**Clinic characteristics**					
BMI, kg/m^2^	23.3 (22.5, 24.1)	23.5 (22.7, 24.3)	22.8 (22.0, 23.6)	22.7 (21.8, 23.5)	0.158
Metabolic syndrome, %	17.1	22.6	11.4	19.3	0.598
Hs-CRP, mg/L	0.7 (0.2, 1.3)	1.0 (0.4, 1.6)	1.3 (0.7, 1.9)	0.6 (−0.1, 1.2)	0.220
Adiponectin, mg/L	7.1 (6.3, 7.9)	6.9 (6.0, 7.7)	7.7 (6.9, 8.6)	7.2 (6.3, 8.2)	0.990
**Daily nutrient intake**					
Total energy intake, kcal/day	1707.3 (1562.1, 1852.5)	1869.7 (1715.5, 2023.8)	2001.9 (1857.6, 2146.2)	2160.2 (1996.0, 2324.4)	<0.001
Total protein intake, g/day	55.9 (50.3, 61.5)	59.8 (53.8, 65.8)	69.3 (63.7, 74.9)	73.1 (66.8, 79.5)	<0.001
Calcium, mg/day	374.8 (326.0, 423.6)	424.0 (372.2, 475.9)	522.6 (474.0, 571.1)	557.1 (501.9, 612.4)	<0.001
Vitamin D, µg/day	9.6 (7.7, 11.4)	9.9 (7.9, 11.9)	13.7 (11.8, 15.6)	12.9 (10.8, 15.0)	0.005
**Other types of vegetables consumption**					
Green-leaf vegetables, g/day	18.5 (11.5, 25.5)	28.4 (21.0, 35.9)	36.3 (29.4, 43.2)	54.2 (46.3, 62.1)	<0.001
Cabbage and Chinese Cabbage, g/day	21.7 (15.5, 27.8)	27.5 (21.0, 34.0)	33.0 (26.9, 39.1)	46.8 (39.8, 53.7)	<0.001
Carrot and pumpkin, g/day	9.9 (6.4, 13.5)	13.3 (9.5, 17.1)	19.1 (15.6, 22.7)	24.3 (20.3, 28.4)	<0.001
Japanese white radish and Turnips, g/day	15.5 (10.5, 20.4)	15.0 (9.8, 20.2)	20.9 (16.0, 25.8)	32.1 (26.6, 37.7)	<0.001
Onion, burdock, lotus root, g/day	19.6 (14.2, 25.0)	24.9 (19.2, 30.7)	30.0 (24.6, 35.3)	43.0 (36.8, 49.1)	<0.001
**Fruit consumption**					
Citrus Fruit, g/day	5.7 (0.7, 10.7)	13.3 (8.0, 18.6)	12.2 (7.2, 17.2)	16.5 (10.9, 22.2)	0.034
Strawberries, persimmon and Kiwifruit, g/day	3.2 (−0.6, 7.1)	6.9 (2.8, 11.0)	6.8 (3.0, 10.6)	12.1 (7.8, 16.5)	0.008
Apple and banana, g/day	17.0 (10.1, 23.9)	22.5 (15.2, 29.9)	25.9 (19.1, 32.8)	39.3 (31.5, 47.1)	0.026

BMI, body mass index; hs-CRP, hig-sensitivity C-reactive protein; MET, metabolic equivalent; PA, physical activity.

^a^Continuous variable without a normal distribution were log-transformed; Continuous variables are expressed as the estimated geometric means (95% confidence intervals) and categorical variables are expressed as percentages.

^b^Linear trends were assessed using ANCOVA for continuous variables and logistic regression analyses for categorical variables.

Table 2 shows the relationships of the frequency of consumption of tomato and tomato product and other types of vegetables at baseline with change in handgrip strength over the 3-year follow-up period. Higher frequency of consumption of tomato and tomato product was associated with reduced decline in handgrip strength after adjustment for all covariates. In model 3, the mean change in handgrip strength among the categories of tomato and tomato product consumption were −3.2 (95% CI, −4.0 to −2.3) for <1 time/week, −2.7 (95% CI, −3.6 to −1.8) for 1 time/week, −1.6 (95% CI, −2.5 to −0.8) for 2–3 times/week, and −1.7 (95% CI, −2.8 to −0.7) for ≥4 times/week (*P* for trend = 0.022). We also conducted a stratified gender-specific analysis, which confirmed that consumption of tomato and tomato product was associated negatively with the change in handgrip strength among males (*P* for trend = 0.028); for females, there was not a negative relationship between consumption of tomato and tomato product and change in handgrip strength (*P* for trend = 0.102) (eTable 1). There were no significant relationships between the consumption of other types of vegetables and changes in handgrip strength.

**Table 2.** Multivariable-adjusted relationships of consumption of different types of vegetables with change in handgrip strength during the 3-year follow-up period

*n* = 259	Number of participants	Mean intake, g/day	Model 1^a^	Model 2^b^	Model 3^c^
Categories of tomato and tomato product consumption					
<1 time/week	70	3.6	−3.3 (−4.2, −2.5)	−3.2 (−4.0, −2.4)	−3.2 (−4.0, −2.3)
1 time/week	62	11.3	−3.0 (−4.0, −2.1)	−2.7 (−3.5, −1.8)	−2.7 (−3.6, −1.8)
2–3 times/week	70	27.5	−1.5 (−2.4, −0.6)	−1.6 (−2.4, −0.8)	−1.6 (−2.5, −0.8)
≥4 times/week	57	64.6	−1.4 (−2.3, −0.4)	−1.8 (−2.8, −0.9)	−1.7 (−2.8, −0.7)
*P* for trend^d^	—	—	<0.001	0.012	0.022
Categories of green-leaf vegetables consumption					
<1 time/week	59	4.1	−3.7 (−4.6, −2.7)	−3.3 (−4.2, −2.4)	−3.1 (−4.1, −2.1)
1 time/week	54	13.0	−2.3 (−3.3, −1.3)	−2.5 (−3.4, −1.6)	−2.4 (−3.4, −1.4)
2–3 times/week	82	32.1	−1.4 (−2.2, −0.5)	−1.5 (−2.2, −0.7)	−1.6 (−2.3, −0.8)
≥4 times/week	64	79.9	−2.3 (−3.2, −1.4)	−2.4 (−3.2, −1.5)	−2.5 (−3.5, −1.4)
*P* for trend^d^	—	—	0.019	0.051	0.305
Categories of cabbage and Chinese cabbage consumption					
<1 time/week	39	5.0	−3.0 (−4.2, −1.8)	−2.6 (−3.7, −1.5)	−2.1 (−3.3, −0.9)
1 time/week	63	12.7	−1.9 (−2.8, −1.0)	−2.1 (−2.9, −1.2)	−1.9 (−2.8, −1.0)
2–3 times/week	109	32.7	−2.4 (−3.2, −1.7)	−2.4 (−3.1, −1.7)	−2.4 (−3.0, −1.7)
≥4 times/week	48	75.8	−2.1 (−3.2, −1.0)	−2.3 (−3.3, −1.3)	−2.9 (−4.2, −1.7)
*P* for trend^d^	—	—	0.408	0.804	0.342
Categories of carrot and pumpkin consumption					
<1 time/week	66	2.5	−3.2 (−4.1, −2.3)	−2.9 (−3.8, −2.1)	−2.8 (−3.8, −1.9)
1 time/week	55	7.4	−2.2 (−3.2, −1.2)	−2.4 (−3.3, −1.4)	−2.4 (−3.3, −1.4)
2–3 times/week	95	18.2	−1.9 (−2.7, −1.2)	−1.9 (−2.6, −1.2)	−1.9 (−2.6, −1.2)
≥4 times/week	43	45.1	−2.0 (−3.1, −0.9)	−2.1 (−3.2, −1.1)	−2.4 (−3.8, −0.9)
*P* for trend^d^	—	—	0.091	0.189	0.554
Categories of Japanese white radish (daikon) and turnips consumption					
<1 time/week	76	3.4	−2.2 (−3.1, −1.3)	−2.3 (−3.1, −1.5)	−2.0 (−2.9, −1.1)
1 time/week	75	11.0	−2.1 (−3.0, −1.3)	−2.3 (−3.1, −1.5)	−2.2 (−3.0, −1.4)
2–3 times/week	78	27.8	−2.7 (−3.5, −1.8)	−2.4 (−3.2, −1.6)	−2.5 (−3.3, −1.7)
≥4 times/week	30	68.4	−2.2 (−3.6, −0.8)	−2.2 (−3.5, −0.9)	−3.0 (−4.6, −1.3)
*P* for trend^d^	—	—	0.824	0.956	0.330
Categories of onion, burdock, lotus root consumption					
<1 time/week	39	3.7	−2.3 (−3.5, −1.1)	−2.3 (−3.4, −1.2)	−2.0 (−3.2, −0.8)
1 time/week	67	11.1	−2.4 (−3.3, −1.5)	−2.6 (−3.4, −1.7)	−2.4 (−3.3, −1.5)
2–3 times/week	91	27.9	−2.5 (−3.3, −1.7)	−2.3 (−3.0, −1.5)	−2.2 (−2.9, −1.4)
≥4 times/week	62	65.1	−2.0 (−2.9, −1.0)	−2.1 (−3.0, −1.2)	−2.7 (−3.7, −1.6)
*P* for trend^d^	—	—	0.701	0.696	0.503

^a^Model 1: Adjusted for age (continuous variable) at baseline.

^b^Model 2: Adjusted for the variables in model 1 and sex (categorical variable), occupation (categorical variable: desk work or not), smoking status (categorical variable: never, former, or current), drinking frequency (categorical variable: 7 times/week, 1–6 times/week and none), PA (continuous variable), sleep duration (categorical variable: 6–8 hours or not), BMI (continuous variable), metabolic syndrome (categorical variable: yes or no), hs-CRP (continuous variable), adiponectin (continuous variable), and handgrip strength at baseline.

^c^Model 3: Adjusted for the variables in model 2 and total energy (continuous variable), total protein (continuous variable), calcium (continuous variable), vitamin D (continuous variable) and mutual other types of vegetables (continuous variable) and fruit consumption (continuous variable: citrus fruit; strawberries, persimmon and kiwifruit; apple and banana) at baseline.

^d^Linear trends were assessed using ANCOVA.

**Figure 1.**
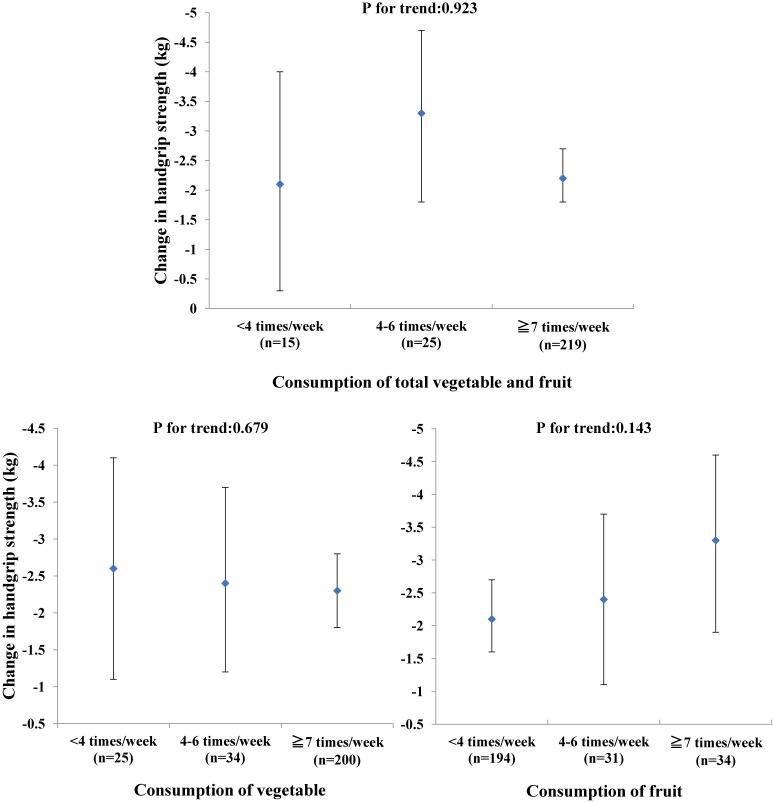
Pooled analysis of relationships of vegetable and fruit consumption with change in handgrip strength among Japanese adults (2008–2011). Means for change in handgrip strength are presented for total vegetable and fruit, vegetable, fruit consumption. The multivariate model that was adjusted for covariates as in Table 2 excluded total vegetable and fruit, vegetable, and fruit consumption.

We also investigated the relationships of different types of fruits consumption and pooled vegetable and fruit consumption with change in handgrip strength. However, the relationships of different types of fruits and pooled vegetable and fruit consumption with reduced decline in handgrip strength were not significant (Table 3 and Figure 1).

**Table 3.** Multivariable-adjusted relationships of consumption of different types of fruits with change in handgrip strength during the 3-year follow-up period

*n* = 259	Number of participants	Mean intake, g/day	Model 1^a^	Model 2^b^	Model 3^c^
Categories of citrus fruit consumption					
0 times/week	112	0.0	−2.2 (−2.9, −1.5)	−2.1 (−2.8, −1.5)	−2.0 (−2.7, −1.3)
<1 time/week	71	6.7	−2.8 (−3.7, −1.9)	−2.6 (−3.4, −1.7)	−2.7 (−3.5, −1.9)
≥1 time/week	76	33.5	−2.0 (−2.9, −1.2)	−2.4 (−3.2, −1.6)	−2.4 (−3.3, −1.6)
*P* for trend^d^	—	—	0.699	0.619	0.486
Categories of strawberries, persimmon and kiwifruit consumption					
0 times/week	146	0.0	−2.4 (−3.0, −1.8)	−2.4 (−2.9, −1.8)	−2.3 (−2.9, −1.7)
<1 time/week	62	6.7	−2.3 (−3.3, −1.4)	−2.4 (−3.3, −1.5)	−2.5 (−3.3, −1.6)
≥1 time/week	51	27.5	−2.1 (−3.1, −1.0)	−2.1 (−3.1, −1.1)	−2.2 (−3.2, −1.2)
*P* for trend^d^	—	—	0.583	0.613	0.855
Categories of apple and banana consumption					
0 times/week	25	0.0	−2.3 (−3.8, −0.8)	−1.9 (−3.3, −0.5)	−1.7 (−3.1, −0.2)
<1 time/week	78	6.6	−2.7 (−3.6, −1.9)	−2.6 (−3.4, −1.9)	−2.5 (−3.3, −1.7)
≥1 time/week	156	39.3	−2.1 (−2.7, −1.5)	−2.2 (−2.8, −1.7)	−2.3 (−2.9, −1.8)
*P* for trend^d^	—	—	0.832	0.651	0.417

^a^Model 1: Adjusted for age (continuous variable) at baseline.

^b^Model 2: Adjusted for the variables in model 1 and sex (categorical variable), occupation (categorical variable: desk work or not), smoking status (categorical variable: never, former, or current), drinking frequency (categorical variable: 7 times/week, 1–6 times/week and none), PA (continuous variable), sleep duration (categorical variable: 6–8 hours or not), BMI (continuous variable), metabolic syndrome (categorical variable: yes or no), hs-CRP (continuous variable), adiponectin (continuous variable), and handgrip strength at baseline.

^c^Model 3: Adjusted for the variables in model 2 and total energy (continuous variable), total protein (continuous variable), calcium (continuous variable), vitamin D (continuous variable) and mutual other types of fruits (continuous variable) and vegetable consumption (continuous variable: tomato and tomato product; green-leaf vegetables; cabbage and Chinese cabbage; carrot and pumpkin; Japanese white radish [daikon] and turnips; onion, burdock, lotus root) at baseline.

^d^Linear trends were assessed using ANCOVA.

## DISCUSSION

This 3-year longitudinal study examined the relationships of the frequency of consumption of different types of vegetables or fruits with change in handgrip strength over time among Japanese adults. These results showed a significant relationship of higher baseline frequency of consumption of tomato and tomato product, but not other types of vegetables and different types of fruits, with reduced decline in handgrip strength, after adjustment for confounding factors. Our findings suggested that consumption of tomato and tomato product could be protective against the decline in skeletal muscle strength associated with aging among Japanese adults.

Results from this longitudinal study support the hypothesis that higher consumption of tomato and tomato product, but not other types of vegetables, could slow the decline in skeletal muscle strength associated with aging. The present findings here are consistent with those of several previous studies. For instance, Kim et al^14^ investigated the relationship of vegetable and fruit consumption with sarcopenia in older adults through a cross-sectional study and found that higher consumption of vegetable and fruit was associated with a lower risk of sarcopenia. Additionally, an interventional study showed that increased consumption of vegetable and fruit may modestly increase handgrip strength in older adults.^15^

Regarding potential mechanisms underlying the relationship of the frequency of consumption of tomato and tomato product with decline in handgrip strength, antioxidants present in tomato may mediate this effect. Skeletal muscles are one of the largest oxygenated tissues, and muscle fibers continuously generate reactive oxygen species with aging.^16^ Oxidative damage is increased when the production of reactive oxygen species increases and/or the antioxidant status decreases. Moreover, according to the cellular constituents affected by the oxidative stress, lipid peroxidation,^17^ protein oxidation,^18^ and DNA damage^19^ will result in abnormalities of cellular structures, leading to cell death. Such cellular decline is believed to be responsible for the degeneration of skeletal muscle strength.^16^ Tomato and tomato product account for more than 85% of all the dietary sources of lycopene, a powerful antioxidant.^20^ Lycopene is a lipid-soluble carotenoid that can interact with the lipid membrane bilayer. It has the ability to scavenge free radicals and quench singlet oxygen^21^; dietary antioxidants are efficient in preventing oxidative damage to lipoproteins.^22^ Antioxidant supplements are reported to reduce oxidative DNA damage^21^ and to improve protein synthesis.^23^ Therefore, dietary lycopene may help delay the decline in skeletal muscle strength.

The present study also revealed that a higher frequency of consumption of tomato and tomato product was associated with higher levels of consumption of trace elements, including vitamin D and calcium. A randomized controlled trial has shown that vitamin D and calcium supplementation was associated with an increase in skeletal muscle strength in older populations.^24^ Vitamin D and calcium intake may enhance nerve conduction and transmission at the neuromuscular junction and, in turn, enhance contraction of skeletal muscles.^25^ However, additional adjustment for dietary intake of vitamin D and calcium in our analysis did not alter our findings.

Alternatively, cooking methods could also explain our findings that higher consumption of tomato and tomato product, but not other types of vegetables, was significantly associated with reduced decline in handgrip strength. In Japan, tomato is most often consumed raw, while other types of vegetables are frequently consumed after cooking. A biochemical study showed that the cooking method not only affects the nutritional composition of food but also the level of available bioactive compounds.^26^ Experimental studies have provided evidence that cooking has a significant destructive effect on the antioxidant activity in food products,^27^^,^^28^ and antioxidants are leached from food to the cooking water^29^ or oil.^30^ Indeed, Danesi et al proposed that, at least for antioxidant activity, raw and fresh vegetable have superior nutritional value compared to cooked vegetable.^27^ Thus, further studies are needed to examine the influence of cooking methods of vegetable on the change in handgrip strength over time.

Although fruit also rich in a large number of phytochemical antioxidants, such as carotenoids and vitamin C, we did not find the significant relationships between the consumption of different types of fruits or total fruit and change in handgrip strength. It is possible that, in our population, fruit consumption was at an insufficient level to show no relationship with handgrip strength (the median consumption of total fruit was 2 times/week). In a mean 2.5-year follow-up study, fruit consumption was not associated with improvement of skeletal muscle strength among community-dwelling older adults, even in populations with an average consumption of 3 portions of fruit/day.^31^

We also conducted analysis regarding the relationships of pooled frequency of vegetable and fruit or vegetable consumption with change in handgrip strength. However, the current study did not show any significant relationships in multivariate analysis. It is possible that the participants in our study who ate tomato and tomato product more frequently could have consumed fewer other types of vegetables or fruits. Further study is needed to clarify the relationships of pooled frequency of vegetable and fruit or vegetable consumption with change in handgrip strength.

We also considered potential confounding factors, including age, BMI, occupation, smoking status, drinking frequency, PA, sleep duration, metabolic syndrome, hs-CRP, adiponectin, consumption of total energy, total protein and handgrip strength at baseline. However, the relationship remained significant, even after adjustment for these confounding factors. Our results suggest that consumption of tomato and tomato product is independently associated with the decline in handgrip strength observed in aging.

There are limitations in the present study. First, due to our observational design, it is difficult to draw a causal relationship. Future studies should focus on confirming causality using interventional or experimental methods. Second, our regional results may not be representative of other populations. Further studies with larger sample sizes are essential to confirm these findings. Third, although the validated BDHQ was used to survey dietary information, there is still some subjectivity in self-reporting. Fourth, the present study did not measure muscle fiber size or muscle mass, both of which are predictive factors for skeletal muscle strength. Therefore, further studies are warranted to assess these two indicators. Fifth, the relatively large number of participants who were not recruited and those who did not return for follow-up might have biased the results; we examined whether there are differences in independent variables between participants and nonparticipants, but there were no significant relationships among them (eTable 2).

In conclusion, this study found that increased frequency of consumption of tomato and tomato product was associated with reduced decline in handgrip strength among Japanese adults, even after consideration of potential confounders. Therefore, this study suggests that consumption of tomato and tomato product may be protective against the decline in skeletal muscle strength associated with aging.

## APPENDIX A. SUPPLEMENTARY DATA

eTable 1. Multivariable-adjusted sex-specific relationships of consumption of tomato and tomato product and other types of vegetables with change in handgrip strength during the 3-year follow-up period

eTable 2. Subject’s baseline characteristics according to handgrip strength with missing values and without missing values at baseline and follow-up period
