# Supplementary material for: High Tomato and Tomato Product Consumption is Protective Against the Decline in Handgrip Strength Among Japanese Adults: The Oroshisho Study
Source: J Epidemiol. 2018 Sep 5;28(9):397–403. doi: 10.2188/jea.JE20170029 (PMC6111105; doi:10.2188/jea.JE20170029)
Supplement: Supplementary file 1 [file je-28-397-s001.pdf]

eTable 1. Multivariable-adjusted sex-specific relationships of consumption of tomato and tomato product and other types of vegetables with change in handgrip strength during the 3-year follow-up period

n=259	Number of participants	Mean intake, g/day	Model 1 [a]	Model 2 [b]	Model 3 [c]
**Males (n=201)**					
**Categories of tomato and tomato product consumption**					
**<1 time/week**	58	3.8	−3.7 (−4.7, −2.6)	−3.6 (−4.6, −2.6)	−3.6 (−4.6, −2.5)
**1 time/week**	53	11.5	−3.3 (−4.4, −2.2)	−3.0 (−4.1, −2.0)	−3.1 (−4.1, −2.0)
**2–3 times/week**	54	28.6	−1.9 (−3.0, −0.8)	−2.0 (−3.0, −1.0)	−2.0 (−3.0, −1.0)
**≥4 times/week**	36	65.1	−1.4 (−2.7, −0.1)	−1.8 (−3.0, −0.5)	−1.8 (−3.2, −0.4)
**P for trend [d]**	—	—	0.002	0.011	0.028
**Categories of green-leaf vegetables consumption**					
**<1 time/week**	49	4.2	−4.1 (−5.3, −3.0)	−3.8 (−4.8, −2.7)	−3.4 (−4.6, −2.3)
**1 time/week**	46	13.3	−2.7 (−3.8, −1.5)	−3.1 (−4.2, −2.0)	−2.8 (−4.0, −1.7)
**2–3 times/week**	61	33.3	−1.6 (−2.6, −0.6)	−1.6 (−2.5, −0.7)	−1.6 (−2.6, −0.7)
**≥4 times/week**	45	82.1	−2.6 (−3.7, −1.4)	−2.6 (−3.7, −1.5)	−3.1 (−4.5, −1.7)
**P for trend [d]**	—	—	0.027	0.042	0.530
**Categories of cabbage and Chinese cabbage consumption**					
**<1 time/week**	28	5.2	−3.9 (−5.4, −2.4)	−3.3 (−4.7, −1.8)	−2.6 (−4.2, −1.0)
**1 time/week**	51	13.2	−2.3 (−3.4, −1.2)	−2.5 (−3.6, −1.5)	−2.3 (−3.4, −1.2)
**2–3 times/week**	90	33.5	−2.7 (−3.5, −1.8)	−2.7 (−3.5, −1.9)	−2.6 (−3.4, −1.8)
**≥4 times/week**	32	76.9	−2.4 (−3.8, −0.9)	−2.4 (−3.7, −1.1)	−3.5 (−5.2, −1.7)
**P for trend [d]**	—	—	0.186	0.440	0.483
**Categories of carrot and pumpkin consumption**					
**<1 time/week**	51	2.6	−3.9 (−5.0, −2.8)	−3.7 (−4.7, −2.6)	−3.5 (−4.6, −2.3)
**1 time/week**	48	7.6	−2.3 (−3.4, −1.1)	−2.9 (−4.0, −1.8)	−2.8 (−4.0, −1.7)
**2–3 times/week**	73	19.0	−2.2 (−3.2, −1.3)	−2.0 (−2.9, −1.2)	−2.0 (−2.9, −1.1)

**≥4 times/week**	29	46.2	−2.3 (−3.8, −0.8)	−2.3 (−3.7, −0.9)	−2.8 (−4.7, −0.9)
**P for trend [d]**	—	—	0.087	0.077	0.465
**Categories of Japanese white radish (daikon) and turnips consumption**					
**<1 time/week**	49	3.6	−3.2 (−4.4, −2.0)	−3.0 (−4.1, −1.9)	−2.6 (−3.8, −1.4)
**1 time/week**	62	11.2	−2.3 (−3.3, −1.3)	−2.6 (−3.6, −1.7)	−2.4 (−3.5, −1.4)
**2–3 times/week**	65	28.7	−2.9 (−3.9, −2.0)	−2.7 (−3.7, −1.8)	−2.7 (−3.7, −1.7)
**≥4 times/week**	25	69.7	−2.0 (−3.6, −0.3)	−2.2 (−3.7, −0.6)	−3.4 (−5.4, −1.4)
**P for trend [d]**	—	—	0.334	0.439	0.519
**Categories of onion, burdock, lotus root consumption**					
**<1 time/week**	28	4.1	−2.9 (−4.5, −1.4)	−2.9 (−4.3, −1.5)	−2.6 (−4.1, −1.0)
**1 time/week**	55	11.4	−2.7 (−3.8, −1.6)	−3.0 (−4.0, −2.0)	−2.8 (−3.9, −1.7)
**2–3 times/week**	77	28.6	−2.8 (−3.8, −1.9)	−2.6 (−3.4, −1.7)	−2.4 (−3.3, −1.6)
**≥4 times/week**	41	65.1	−2.2 (−3.5, −0.9)	−2.4 (−3.5, −1.2)	−3.1 (−4.5, −1.7)
**P for trend [d]**	—	—	0.500	0.481	0.746
**Females (n=58)**					
**Categories of tomato and tomato product consumption**					
**<1 time/week**	12	2.6	−1.7 (−3.0, −0.4)	−2.2 (−3.9, −0.6)	−2.6 (−4.5, −0.7)
**1 time/week**	9	10.1	−1.4 (−2.9, 0.1)	−1.0 (−2.6, 0.6)	−1.5 (−3.4, 0.4)
**2–3 times/week**	16	23.9	0.0 (−1.1, 1.1)	0.0 (−1.2, 1.2)	−0.1 (−1.4, 1.3)
**≥4 times/week**	21	63.7	−1.4 (−2.4, −0.4)	−1.3 (−2.4, −0.1)	−0.7 (−2.0, 0.6)
**P for trend [d]**	—	—	0.383	0.261	0.102
**Categories of green-leaf vegetables consumption**					
**<1 time/week**	10	3.7	−1.3 (−2.7, 0.2)	−1.0 (−2.6, 0.6)	−1.7 (−3.9, 0.6)
**1 time/week**	8	11.2	−0.4 (−2.0, 1.2)	−0.4 (−2.2, 1.4)	−0.8 (−3.0, 1.4)
**2–3 times/week**	21	28.3	−0.6 (−1.6, 0.4)	−0.6 (−1.7, 0.5)	−0.7 (−1.9, 0.5)
**≥4 times/week**	19	74.8	−1.8 (−2.8, −0.7)	−1.9 (−3.1, −0.7)	−1.3 (−2.9, 0.3)
**P for trend [d]**	—	—	0.562	0.394	0.818
**Categories of cabbage and Chinese cabbage consumption**					
**<1 time/week**	11	4.5	−0.6 (−1.9, 0.8)	−0.8 (−2.3, 0.7)	−1.1 (−3.2, 1.0)
**1 time/week**	12	10.7	−0.3 (−1.6, 1.0)	−0.1 (−1.5, 1.4)	−0.3 (−2.1, 1.5)
**2–3 times/week**	19	28.8	−1.4 (−2.4, −0.3)	−1.2 (−2.4, −0.1)	−1.3 (−2.7, 0.0)
**≥4 times/week**	16	73.7	−1.7 (−2.8, −	−1.8 (−3.1, −	−1.3 (−3.1, 0.5)

			0.5)	0.5)	
**P for trend [d]**	—	—	0.128	0.187	0.741
**Categories of carrot and pumpkin consumption**					
**<1 time/week**	15	2.2	−0.8 (−2.0, 0.4)	−0.7 (−2.1, 0.7)	−1.7 (−3.6, 0.3)
**1 time/week**	7	6.1	−1.7 (−3.4, 0.1)	−0.8 (−2.8, 1.2)	−1.0 (−3.3, 1.2)
**2–3 times/week**	22	15.8	−0.8 (−1.8, 0.2)	−1.2 (−2.3, −0.1)	−1.4 (−2.7, −0.1)
**≥4 times/week**	14	42.8	−1.4 (−2.7, −0.2)	−1.4 (−2.9, 0.0)	0.1 (−2.3, 2.4)
**P for trend [d]**	—	—	0.693	0.439	0.379
**Categories of Japanese white radish (daikon) and turnips consumption**					
**<1 time/week**	27	3.0	−0.4 (−1.2, 0.5)	−0.4 (−1.3, 0.5)	−0.4 (−1.5, 0.8)
**1 time/week**	13	9.6	−1.4 (−2.6, −0.2)	−1.3 (−2.7, 0.1)	−1.5 (−3.1, 0.1)
**2–3 times/week**	13	23.7	−1.4 (−2.7, −0.2)	−1.3 (−2.6, 0.1)	−1.3 (−3.1, 0.5)
**≥4 times/week**	5	62.1	−3.1 (−5.1, −1.2)	−3.7 (−5.9, −1.5)	−3.2 (−6.5, 0.0)
**P for trend [d]**	—	—	0.014	0.013	0.154
**Categories of onion, burdock, lotus root consumption**					
**<1 time/week**	11	2.8	−0.9 (−2.3, 0.6)	−0.9 (−2.6, 0.7)	−1.5 (−3.8, 0.7)
**1 time/week**	12	9.8	−0.9 (−2.2, 0.5)	−0.9 (−2.4, 0.6)	−1.1 (−2.8, 0.7)
**2–3 times/week**	14	23.9	−0.7 (−2.0, 0.5)	−0.9 (−2.3, 0.5)	−0.7 (−2.4, 1.0)
**≥4 times/week**	21	65.2	−1.5 (−2.5, −0.5)	−1.3 (−2.5, −0.2)	−1.1 (−2.6, 0.4)
**P for trend [d]**	—	—	0.508	0.716	0.711

[a] Model 1: Adjusted for age (continuous variable) at baseline.

[b] Model 2: Adjusted for the variables in model 1 and occupation (categorical variable: desk work or not), smoking status (categorical variable: never, former, or current), drinking frequency (categorical variable: 7 times/week, 1-6 times/week and none), PA (continuous variable),sleep duration (categorical variable: 6-8 hours or not), BMI (continuous variable), metabolic syndrome (categorical variable: yes or no), hs-CRP (continuous variable), adiponectin (continuous variable) and handgrip strength (continuous variable) at baseline.

[c] Model 3: Adjusted for the variables in model 2 and total energy (continuous variable), total protein (continuous variable), calcium (continuous variable), vitamin D (continuous variable) and mutual other types of vegetables (continuous variable), fruit consumption (continuous variable: citrus fruit; strawberries, persimmon and kiwifruit; apple and banana) at baseline.

[d] Linear trends were assessed using ANCOVA.

eTable 2. Subject's baseline characteristics according to handgrip strength with missing values and without missing values at baseline and follow-up period

	**Baseline data with missing handgrip strength**	**Baseline data without missing handgrip strength**	**Follow-up data with missing handgrip strength**	**Follow-up data without missing handgrip strength**
**Demographic characteristics**				
Sex (men), %	76.9	75.7	73.5	79.7
Age, years	46.8 (46.0, 47.7)	45.6 (44.7, 46.4)	46.4 (45.5, 47.2)	46.0 (45.1, 46.9)
Occupation (desk work), %	43.1	47.2	46.0	44.1
**Lifestyle characteristics**				
Smoking status				
Current, %	44.8	44.9	45.4	44.1
Former, %	11.5	12.7	11.7	12.7
Drinking frequency				
7 times/week, %	28.2	28.1	26.8	29.7
≦6 times/week, %	50.8	51.0	51.4	50.3
PA, MET·h·week^{-1}	37.0 (30.5, 43.5)	38.4 (32.2, 44.6)	38.2 (32.2, 44.3)	37.5 (30.8, 44.2)
Sleep duration (6-8 h), %	56.2	53.9	54.3	55.8
**Clinic characteristics**				
BMI, kg/m^2	23.3 (23.0, 23.6)	23.1 (22.8, 23.4)	23.3 (23.0, 23.5)	23.1 (22.8, 23.5)
Metabolic syndrome, %	20.7	17.5	18.8	19.4
High-sensitivity CRP, mg/L	1.0 (0.7, 1.3)	1.0 (0.7, 1.3)	1.2 (0.9, 1.4)	0.8 (0.5, 1.2)
Adiponectin, mg/L	7.3 (7.0, 7.7)	7.4 (7.1, 7.7)	7.5 (7.2, 7.8)	7.2 (6.9, 7.5)
**Daily nutrient intake**				
Total energy intake, kcal/day	1852.5 (1799.8, 1905.1)	1904.5 (1853.4, 1955.6)	1836.6 (1787.0, 1886.2)	1930.8 (1876.3, 1985.2)
Total protein intake, g/day	63.3 (61.0, 65.5)	64.9 (62.8, 67.1)	63.1 (61.0, 65.2)	65.5 (63.2, 67.7)
Calcium, mg/day	479.3 (458.2, 500.4)	484.2 (463.8, 504.7)	480.1 (460.2, 499.8)	485.0 (463.2, 506.8)
Vitamin D, µg/day	11.7 (10.9, 12.5)	12.1 (11.3, 12.9)	11.7 (11.0, 12.5)	12.2 (11.4, 13.0)
**Other types of vegetables consumption**				
Tomato and tomato products, g/day	24.6 (22.3, 26.9)	26.3 (24.0, 28.5)	26.4 (24.2, 28.6)	24.3 (22.0, 26.7)

Green-leaf vegetables, g/day	32.8 (30.0, 35.6)	32.8 (30.1, 35.5)	32.0 (29.4, 34.7)	33.7 (30.8, 36.6)
Cabbage and Chinese Cabbage, g/day	31.1 (28.7, 33.5)	32.4 (30.1, 34.8)	31.2 (28.9, 33.5)	32.5 (30.1, 35.0)
Carrot and pumpkin, g/day	15.3 (14.0, 16.6)	16.3 (15.0, 17.6)	15.2 (14.0, 16.4)	16.5 (15.2, 17.8)
Japanese white radish and Turnips, g/day	19.0 (17.2, 20.9)	20.4 (18.6, 22.2)	19.0 (17.2, 20.7)	20.7 (18.8, 22.5)
Onion, burdock, lotus root, g/day	28.1 (26.0, 30.2)	28.8 (26.8, 30.8)	27.5 (25.5, 29.4)	29.6 (27.5, 31.8)
**Fruit consumption**				
Citrus Fruit, g/day	11.1 (9.5, 12.8)	11.9 (10.3, 13.5)	11.1 (9.6, 12.7)	12.0 (10.3, 13.7)
Strawberries, persimmon and Kiwifruit, g/day	6.4 (5.0, 7.8)	8.3 (6.9, 9.7)	7.5 (6.2, 8.8)	7.3 (5.8, 8.7)
Apple and banana, g/day	26.5 (23.8, 29.2)	25.9 (23.3, 28.5)	26.0 (23.5, 28.5)	26.5 (23.7, 29.2)
